# Pharmacokinetic Variability in Pre-Clinical Studies: Sample Study with Abiraterone in Rats and Implications for Short-Term Comparative Pharmacokinetic Study Designs

**DOI:** 10.3390/pharmaceutics14030643

**Published:** 2022-03-15

**Authors:** Jana Královičová, Aleš Bartůněk, Jiří Hofmann, Tomáš Křížek, Petr Kozlík, Jaroslava Roušarová, Pavel Ryšánek, Martin Šíma, Ondřej Slanař

**Affiliations:** 1 Department of Pharmacology, First Faculty of Medicine, Charles University and General University Hospital in Prague, Albertov 4, 12800 Prague, Czech Republic; ales.bartunek@synavia.com (A.B.); jaroslava.rousarova@lf1.cuni.cz (J.R.); pavel.rysanek@lf1.cuni.cz (P.R.); martin.sima@lf1.cuni.cz (M.Š.); ondrej.slanar@lf1.cuni.cz (O.S.); 2 Zentiva k.s., U Kabelovny 130, 10237 Prague, Czech Republic; jiri.hofmann@zentiva.com; 3 Department of Analytical Chemistry, Faculty of Science, Charles University, Hlavova 8, 12800 Prague, Czech Republic; tomas.krizek@natur.cuni.cz (T.K.); petr.kozlik@natur.cuni.cz (P.K.)

**Keywords:** abiraterone, variability, pharmacokinetics, cross-over design, rat, in vivo study

## Abstract

One of the major concerns for all in vivo experiments is intra- and inter-subject variability, which can be a great source of inaccuracy. The aim of this study is, therefore, to estimate the ability of parallel vs. cross-over design studies in order to describe the relative pharmacokinetic performance of the studied drug formulations. We analyzed the data from a drug development program that examined the performance of innovative abiraterone acetate formulations against the identical reference product in three stages. In stages 1–3, groups A–F were dosed with the reference product once in a parallel manner. Stage 4 was performed to evaluate the intra-individual variability (IIV) by repeated administration of the reference product to the same animals. Although the geometric mean (90% CI) values of abiraterone AUC_last_ in groups A–F were similar to the IIV group (24.36 (23.79–41.00) vs. 26.29 (20.56–47.00) mg/mL·min·g), the results generated in the isolated parallel groups provided imprecise estimates of the true AUC_last_ values ranging from 9.62 to 44.62 mg/mL·min·g due to chance. Notably, in 4 out of 15 possible pair comparisons between the parallel groups, the confidence intervals did not include 100%, which is the true ratio for all comparisons tested after identical formulation administration to all groups. A cross-over design can significantly improve the methodology in short-term comparative pre-clinical pharmacokinetic studies, and can provide more precise and accurate results in comparison to more traditional pre-clinical study designs.

## 1. Introduction

Pre-clinical pharmacokinetic (PK) studies play a key role in several stages of pharmaceutical research, as they are currently an irreplaceable source of basic pharmacokinetic data, and they also allow a relationship to be established between exposure and drug-induced toxicity (toxicokinetics) before a new molecule is administered to humans [1,2]. Furthermore, animal studies are sometimes needed during the development of innovative drug formulations in order to confirm the performance of the drug formulation, estimated from in vitro and modelling studies [3].

Clinical pharmacokinetic trials currently have a rather sophisticated methodology, which is guided by major regulatory agencies worldwide. The methodology of clinical studies has been cultivated for a long time to deliver reliable, accurate and precise data, while minimizing study subjects’ exposure to test compounds. Therefore, the methodology of a clinical pharmacokinetic study is inherently dependent on the hypothesis tested and the aim of the study.

On the other hand, relatively little attention has been paid to the optimal ways of performing the pre-clinical pharmacokinetic studies. When planning these studies, parallel design is usually adopted [4,5]. The limited sampling strategy has also gained popularity in recent years, as it enables pharmacokinetic data to be obtained in a simplified model [6,7]. However, these approaches are often chosen without further considerations and irrespective of the aim of the study.

One of the major concerns for all in vivo experiments is intra- and inter-subject variability, which can be a great source of inaccuracy. Gender, age, weight, hormonal status and eating habits are just a few examples of the factors which introduce variability into the study data [8]. Actual status of the gastrointestinal system, (e.g., gastric and intestinal pH, gastric emptying and intestinal transit, bacterial colonization or surface area [9,10,11]), renal function, hepatic abundance and drug transporters further contribute to the actual fate of a compound in the body [12]. Furthermore, genetic polymorphisms within the genes which encode for drug-metabolizing enzymes or transporters may further substantially affect drug absorption, distribution, or elimination.

As a result, it is almost impossible to ensure the same inner and outer conditions for each subject enrolled in a study. Previous experiments with mice have shown that animals act differently across laboratories despite strict standardization [13]. It has also been demonstrated that mice phenotypes can fluctuate to the extent that the obtained results may differ between batches [14].

We hypothesized that to obtain precise and accurate results during pre-clinical pharmacokinetic studies aimed at comparing drug formulation PK performance, a parallel study design may not be appropriate, and a cross-over design of the pharmacokinetic experiment that limits the consequences of inter-subject variability should be chosen.

Therefore, our aim was to estimate the ability of parallel vs. cross-over design studies to describe the relative pharmacokinetic performance of the studied drug formulations.

## 2. Materials and Methods

### 2.1. Materials

For anesthesia, isoflurane (IsoFlo 250 mL, Zoetis/Pfizer, Prague, Czech Republic), ketamine (Narkamon 100 mg/mL inj sol, Bioveta, Ivanovice na Hané, Czech Republic), and xylazine (Rometar 20 mg/mL inj sol, Bioveta, Ivanovice na Hané, Czech Republic) were used. Amoxicillin with clavulanic acid (Synulox RTU inj 100 mL, Zoetis/Pfizer, Prague, Czech Republic) was used as a perioperative antibiotic, ketoprofen (Ketodolor inj 100 mL, LeVet Pharma b.v., Oudewater, The Netherlands) as an analgesic, and enoxaparin (Clexane inj 4000 IU/0.4 mL, Sanofi-Aventis, Prague, Czech Republic) and heparin (Heparin Léčiva inj 1 × 10 mL/50KU, Zentiva, Prague, Czech Republic) as anticoagulants. Surgical skin glue was obtained from Henry Schein (Brno, Czech Republic). The reference formulation of abiraterone acetate (Zytiga, Janssen-Cilag Spa, Latina, Italy) was provided by Zentiva, k.s. (Prague, Czech Republic). Before dosing, the tablets were crushed and the powder was placed into gelatin capsules containing 4.2 mg of abiraterone acetate.

### 2.2. Animals

Male Wistar rats were purchased from Velaz (Prague, Czech Republic). They were kept under standard conditions with a 12 h light–dark cycle, 22 ± 2 °C temperature, and 50 ± 10% relative humidity. They had ad libitum access to water and a standard granulated diet, with the exception of 4 h before and after dosing with abiraterone acetate. The rats were treated in compliance with the Guiding Principles for the Use of Animals at Charles University, First Faculty of Medicine, and all measures were taken to minimize animal suffering. The experimental animal project was approved by the Ministry of Education, Youth and Sports, Czech Republic (MSMT-9445/2018-8).

### 2.3. Experimental Design and Procedures

We analyzed the data from a drug development program that examined the performance of innovative abiraterone acetate formulations against the identical reference product (Zytiga, Janssen-Cilag Spa, Latina, Italy) in 3 stages. Stage 4 was performed to evaluate the intra-individual variability by repeated administration of the reference product to the same animals. For more detailed information about each stage, see Table 1.

Each stage was conducted in a randomized, single-dose, single-center, laboratory-blinded, two-period, cross-over design comparing bioavailability between the test and reference formulations. For the present analysis, animals that were dosed with the reference product in stages 1–3 are considered as individual groups A–F (*n* = 24). These animals were administered the reference product on one occasion only, which allows for a parallel comparison of the reference product bioavailability between the groups A–F.

In stage 4, the identical reference product was administered to animals (*n* = 6) in both periods in order to investigate the intra-individual variability of abiraterone acetate in a single-dose, single-center, laboratory-blinded, two-period, cross-over design. The group of animals dosed in Stage 4 is labelled as the intra-individual variability (IIV) group.

Prior to dosing, the rats underwent a surgery where *A. carotis* was cannulated with catheters made of medical-grade polyurethane (1.9-3Fr, Instech Laboratories, Plymouth Meeting, PA, USA). Before the surgery, the rats were anesthetized by isoflurane (2.5–5%) following xylazine (5 mg/kg, i.m.) and ketamine (100 mg/kg, i.m.). Amoxicillin with clavulanic acid (140/35 mg/kg, s.c.) was applied to prevent infection during the surgery and ketoprofen (6 mg/kg, s.c.) was used to minimize post-surgery pain. Catheters were flushed with physiological saline (200 μL), heparin (50 μL), and sealed with heparinized glycerol every day after surgery to avoid clogging.

After three days, the rats were randomly placed into groups and a formulation containing abiraterone acetate was administered via oral gavage together with 1 mL of water. To reach a fasted state, access to food was restricted between 4 h before and 4 h after dosing. After drug dosing via gavage, blood samples (100 μL) were collected for 7 h (pre-dose, 0.5, 1, 1.5, 2, 2.5, 3, 4, 5 and 7 h). Blood withdrawn was replaced by physiological saline (100 μL), and catheters were flushed by heparinized saline (1250 IU/mL) and sealed by heparinized glycerol. Centrifugation of blood samples was performed (4500× *g*, 4 °C, 10 min) and obtained serum aliquots were stored at −80 °C until further analysis. A washout period of at least forty-eight hours was kept between study periods, based on the previously reported half-life in rats (so that there are at least five half-lives between the periods) [15]. Sufficient length of the washout was confirmed by measuring plasma levels of abiraterone below LLOQ before starting the second period.

### 2.4. Analytical Methods

For the determination of abiraterone, plasma samples were processed as follows. In 25 µL of plasma, proteins were precipitated by the addition of 100 µL of acetonitrile (containing 32 ng/mL abiraterone-d4 as an internal standard). The mixture was vortexed and centrifuged at 9800× *g* for 10 min. The supernatant was injected into the UHPLC-MS/MS system. Nexera X3 UHPLC coupled with Triple Quad 8045 MS (Shimadzu, Kyoto, Japan) was used with Kinetex EVO C18 column, 100 × 2.1 mm, 1.7 µm particles (Phenomenex, Torrance, CA, USA), thermostated at 40 °C. The mobile phase (A: 0.1% formic acid in deionized water, B: acetonitrile) was pumped in at a flow rate of 0.35 mL/min, and the following gradient program was applied (min/% B) 0/30, 1.5/90, 3.0/90, 3.5/30, 6.0/30. The autosampler temperature was maintained at 10 °C and the sample injection volume was 2 µL. Effluent from the column was directed to the MS ion source between 2.6 and 3.8 min only. For the rest of the time, the effluent was directed to the waste. The MS was operated with positive electrospray ionization, with ion source settings as follows: a nebulizing gas flow of 3 L/min, a heating gas flow of 10 L/min, an interface temperature of 300 °C, a desolvation line temperature of 250 °C, a heat block temperature of 400 °C, and a drying gas flow of 10 L/min. A multiple reaction monitoring (MRM) mode was used for abiraterone quantification. An MRM transition of 350.3 > 156.1 (Q1 pre-bias—17 V, Q3 pre-bias—27 V, collision energy—57 V) was monitored for abiraterone and transition 354.3 > 160.1 (Q1 pre-bias—17 V, Q3 pre-bias—30 V and collision energy—57 V) was monitored for abiraterone-d4. The method was validated concerning linearity, detection limit, accuracy, precision, recovery, selectivity, and matrix effects. A seven-point calibration curve was constructed using the analyte-to-internal standard peak area ratio. Weighted least-squares linear regression (1/x^2^ weighting factor) was used. The developed method was linear in the range of 0.5–600 mg/mL (R^2^ > 0.9996). The detection limit was 0.03 ng/mL (determined as a concentration providing a signal corresponding to 3.3 times blank matrix baseline noise), which was sufficient for the determination of abiraterone in plasma samples. The accuracy expressed as the relative error in % was within ±7.1%. Inter-day and intra-day precision expressed as relative standard deviations in % were between 2.4 and 5.2%. The selectivity of the method was assessed by analysis of six different plasma samples in the scan MS mode. No interfering compounds appeared within the retention time window of abiraterone. It should be noted that the method uses tandem MS in the MRM mode, which further increases the selectivity. Recovery of the method was checked by a comparison between the abiraterone concentration found in a plasma sample spiked with the standard before precipitation of proteins and the concentration found in a plasma sample spiked after precipitation of proteins, at three concentration levels (1, 50 and 250 ng/mL). The matrix effect was evaluated with six different plasma samples spiked with abiraterone standard after precipitation of proteins at two concentration levels (1 and 100 ng/mL). Abiraterone concentrations found in the plasma samples were between 80 and 108% of the concentration found in the spiked 80% acetonitrile. Calibration was performed every day and quality control samples were injected after every seven samples.

### 2.5. Data Analysis and Statistics

Phoenix WinNonlin^®^ 8.3 (Certara, Princeton, NJ, USA) was used to analyze statistics and pharmacokinetics. For AUC_last_, natural logarithmic transformation was used for all statistical inference. For AUC_last_, non-compartmental analysis using linear trapezoidal rule with linear interpolation was employed. For parallel comparisons of groups A–F, the two-one-sided t-tests rely on the assumption that the observations for the groups come from distributions that have equal variances. This assumption is taken since an identical reference formulation was administered to all groups. For the cross-over comparison in the paired design in stage 4, the ANOVA model with the fixed effects subject and period was used. The residual variance from the model was used to construct a 90% confidence interval (CI). For all pharmacokinetic calculations, actual sampling times and ln-transformed concentration data normalized by animal weight were used. All figures and histograms were constructed using GraphPad Prism 9.3.1 (GraphPad Software, San Diego, CA, USA).

### 2.6. Simulation Methods

All simulations were performed in R [16], version 4.0.2, by using the Mersenne Twister algorithm for random number generation. In brief, individual test-to-reference ratios were simulated on the basis of log-normal distribution, where the simulated geometric means ratio (GMR) was 1 and the coefficient of variation was 0.73 and 0.96 for cross-over and parallel design, respectively. These variations were observed in our program for the reference formulation Zytiga. In the case of parallel design, homoscedasticity and balanced treatment groups were respected. The total sample size corresponded to 24 for both designs. For each design, 10,000 simulations were conducted, and the proportion of ratios falling within the standard bioequivalence acceptance range 0.8 to 1.25 was expressed in percentage.

## 3. Results

**Figure 1:**
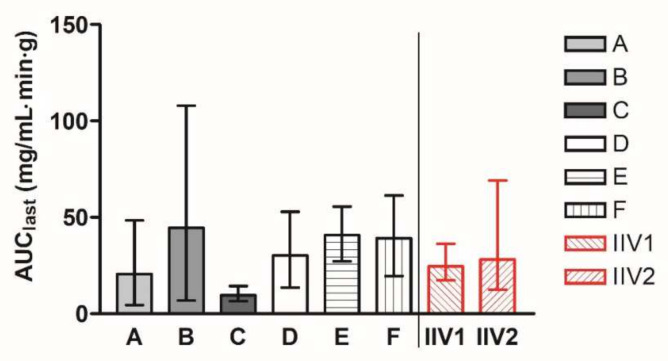
Geometric mean AUC_last_ (90% CI) of individual parallel groups A–F within the program with abiraterone acetate reference product in fasted state. To the right of the division line, AUC_last_ values of group IIV are presented for periods 1 and 2 separately.

In stages 1–3, groups A–F were dosed with the reference product once in a parallel manner. The geometric mean AUC_last_ values of the groups A–F ranged from 9.62 to 44.62 mg/mL·min·g. In stage 4, the group labelled IIV was administered with the same reference product in two periods, described as IIV1 and IIV2. The geometric means of AUC_last_ values are presented for all the groups in Figure 1. The geometric mean (90% CI) AUC_last_ value of groups A–F was 24.36 (23.79–41.00) mg/mL·min·g, and the geometric mean (90% CI) AUC_last_ value of IIV groups was 26.29 (20.56–47.00) mg/mL·min·g.

**Figure 2:**
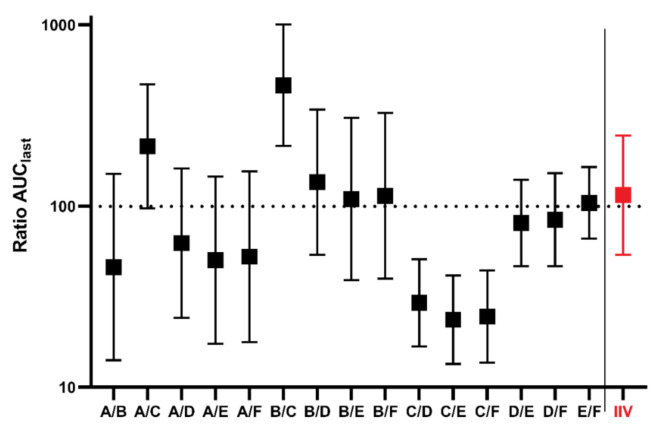
Results of bioequivalence evaluations, and AUC_last_ ratios with 90% CI between groups A–F in parallel design. To the right of the division line is the group IIV AUC_last_ ratio to compare periods from cross-over design. Log scale was used on y axis.

All possible parallel comparisons between groups A–F were calculated, as well as the comparison of period 2 against period 1 for the IIV study. AUC_last_ ratios and 90% CI are presented in Figure 2.

**Figure 3:**
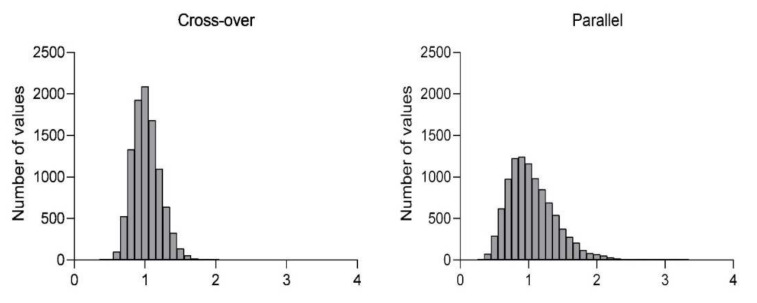
Histograms showing distribution of the resulting ratios in cross-over and parallel designs.

Simulations of the probability that the resulting ratio would fall into the range of 80–125% (standard bioequivalence acceptance range), based on the observed inter-individual variability from groups A–F or IIV in parallel or cross-over design and a sample size of 24 animals, are summarized in Table 2. Histograms showing the distribution of the resulting ratios in cross-over and parallel designs are presented in Figure 3.

## 4. Discussion

When any pre-clinical study is being planned or performed, the 3R principles must be followed. These principles include a reduction in animal use, a replacement of other methods whenever feasible, and minimized pain felt by the animals to the greatest possible extent [17]. In parallel with the 3R principles, more attention has been paid in recent years to the design of pre-clinical pharmacokinetic studies [18,19]. Some attempts to standardize the procedures used in these studies are emerging to improve the quality of the obtained data [3,20]. Our work was conducted to elaborate on the impact of inter- and intra-subject variability within comparative pre-clinical studies, which is an important factor that may substantially affect the obtained results. Abiraterone has been selected as a model compound belonging to BCS class IV, which is a class with reported high inter- as well as intra-animal pharmacokinetic variability [9].

Our analysis indicates that, although the geometric mean (90% CI) values of abiraterone AUC_last_ in the parallel groups A–F were similar to the IIV group (24.36 (23.79–41.00) vs. 26.29 (20.56–47.00) mg/mL·min·g) after the administration of the identical reference formulation, the results generated in the isolated parallel groups provided likely imprecise estimates of the true AUC_last_ values ranging from 9.62 to 44.62 mg/mL·min·g only due to chance. The differences between individual groups were extensive, with a maximum value of geometric means more than 4 times higher than the minimum value. Notably, in 4 out of 15 possible pair comparisons between the parallel groups, the confidence intervals did not include 100%, which is the true ratio for all comparisons tested after identical formulation administration to all groups. That means that in 27% of the studies, identical formulations would be claimed to provide different absorption characteristics compared to the reference formulation. This observation represents a practical example of false positive results that may be generated in studies in parallel design with a limited number of animals enrolled, where it is not feasible to keep all factors standardized and balanced.

It has been described that, despite maximum efforts to standardize the conditions of animal experiments, there is still high variability between various batches in parallel design [13]. Within our work, the same conditions were maintained for all rats throughout their housing and experiments, and identical methodology, analysis, and other processes have been applied.

However, there was still high variability in response between the parallel groups, which can be explained by differences between the subjects and their behaviors: individual genetic background, susceptibility to stress, eating habits that may differ even when all rats are fed the same way, physical activity, and the number of physiological conditions. Many of these factors cannot be measured or controlled during the experiments.

Stage 4 of our program was designed as a cross-over study to investigate intra-subject variability. In this study, all animals received the same formulation in both periods, and the extent of absorption (AUC_last_) from period 2 was compared to period 1 in a paired design. There was no huge difference between the periods, showing that the intra-subject variability was rather low. The coefficient of variability (CV) decreased from 96% in the parallel setting to 73% in the cross-over design. A decrease in variability of this magnitude is a significant factor for the sample size calculation of any planned experiment. For example, to test the significance of a 1.5-fold difference between groups with 80% (β = 0.2) power and α = 0.05, a minimal sample size of 116 and 66 probands was estimated when the CV is 96% and 73%, respectively. The use of a cross-over design would, therefore in such a case, allow the number of animals to be reduced by almost one half.

Additionally, for both parallel and cross-over design, simulations of probability to obtain accurate results confirming equivalence were created. For both designs, the same sample size (*n* = 24) was tested, with the CV obtained from our current work presented. The results show that 76% of runs generated ratios within 80–125% in cross-over design. In the case of parallel design with the same total number of animals, the number of ratios within the 80–125% range falls to 49%. In other words, for comparative studies of this size, there is almost 80% probability of achieving the correct conclusion of similar PK performance between both administrations of identical formulation, while only 50% of studies will generate the same true conclusion in the parallel group design.

Our observation corresponds to the known consequences of variability in human comparative pharmacokinetic studies. For human comparative studies, cross-over design has been recommended as the gold-standard methodology by both the Food and Drug Administration (FDA) and the European Medicines Agency (EMA) for several decades [21,22,23].

The cross-over design brings an indisputable advantage, as a smaller sample size is sufficient to provide reliable results because each subject serves also as its own control. In such a design, a large component of variability that is related to inter-individual differences is eliminated. The choice of such a design in comparative pre-clinical study also fits in with attempts to minimize the number of experimental animals (e.g., the Animal Welfare Act originally from 1966 [17] or Directive 2010/63/EU of the European Parliament and of the Council from 2010 [24]). On the other hand, there is a disadvantage to the cross-over methodology in small animals such as rats, i.e., the need for mature surgical techniques and advanced peri-operative care of the animals in order to minimize blood loss and maintain the animals in good overall status throughout the study.

Contrary to our findings, Daublain et al. reported the rather surprising observation that intra-animal exposures were found to be more variable than inter-animal exposures in their analysis of PK studies with more than 16,000 research compounds [9]. However, this observation is likely to be a consequence of the long duration of the PK studies included in the analysis by Daublain et al., such as toxicokinetic studies, which also introduce the factor of rapid ontogenesis in rats likely to override a single-time-point variability. Therefore, our findings only apply to short-term animal experiments, where the ontogenetic changes of animals do not substantially affect the study animals.

## 5. Conclusions

To conclude, the traditional approach to comparative pre-clinical pharmacokinetic studies brings neither the required accuracy nor precision, if the 3R principles are followed and the number of animals is kept acceptably small. Adopting a cross-over design is one solution to ensure a high quality of data while reducing the number of animals needed for the study. There are a few technical disadvantages of this more complicated study design, especially the need for more advanced care of the animals and the necessity to minimize the blood sample volumes. Despite this, a cross-over design can significantly improve the methodology in short-term comparative pre-clinical pharmacokinetic studies, and can provide more precise and accurate results in comparison to more traditional pre-clinical study designs.

## Figures and Tables

**Table 1:** Summary of formulations administered to rats and number of rats enrolled into each stage and group. R–reference formulation, T1–T3—innovative formulations.

Stage	Group	Formulations	Number of Rats
1	A	R/T1	4
	B	R/T1	4
2	C	R/T2	6
	D	R/T2	4
3	E	R/T3	3
	F	R/T3	3
4	IIV	R/R	6

**Table 2:** Parameters used in simulations with calculated probability that the resulting ratio would fall into the range of 80–125%. CV: intra-subject for cross-over, total for parallel.

	Cross-Over	Parallel
Simulations	10,000	10,000
Sample size	24	24
CV	73%	96%
Ratios 80–125%	76%	49%

## Data Availability

Data supporting the findings of this study are available from the corresponding author upon reasonable request.
